# Targeted coagulation of large stalk vessels in giant pedunculated colorectal polyp: is endoscopic submucosal dissection the new way to go?

**DOI:** 10.1055/a-2113-9626

**Published:** 2023-07-27

**Authors:** Lucille Quénéhervé, Thomas Grainville, Rubeshen Arnachellum, Mathieu Pioche, Tony Michel, Jérémie Jacques, Timothée Wallenhorst

**Affiliations:** 1 Gastroenterology Department, University Hospital of Brest, Brest, France; 2 LaTIM, UMR 1101, Brest University Hospital, Brest, France; 3 Department of Endoscopy and Gastroenterology, Centre Hospitalier Universitaire Pontchaillou, Rennes, France; 4 Department of Endoscopy and Hepatogastroenterology, Pavillon L, Edouard Herriot Hospital, Lyon, France; 5 Department of Endoscopy and Gastroenterology, Centre Hospitalier Universitaire Dupuytren, Limoges, France

Resection of giant pedunculated colorectal polyps can be tricky to achieve owing to the significant risk of bleeding. The European Society of Gastrointestinal Endoscopy therefore recommends injection of diluted adrenalin and/or mechanical hemostatic prevention, such as the use of a detachable snare in cases of head diameter > 20 mm and/or stalk > 10 mm 1 . However, when the head of the polyp is > 25 mm, it may be difficult to enclose it, leading to a significant risk of incomplete resection.

Endoscopic submucosal dissection (ESD) is feasible in this indication 2 and furthermore allows targeted preventive hemostasis of large submucosal vessels, as previously shown in colonic angiodysplasia 3 .

A 65-year-old man was referred with a large polyp in the left colon. Endoscopic examination revealed a giant pedunculated adenoma with a 40-mm head obstructing the lumen. The 15-mm-long, broad-based stalk occupied one-third of the colonic circumference. Large vessels were visible in transparency in the stalk. Placement of a detachable snare prior to endoscopic mucosal resection was deemed difficult because of the bulky head. ESD enabled targeted and safe hemostasis ( Video 1 ) of several large vessels, which were successively identified, isolated, coagulated with hemostatic forceps, and cut during the procedure ( Fig. 1 , Video 1 ). The defect was closed using clips. The polyp ensnared in a retrieval net was extracted using a bivalve metal Halle dilator, which is used in proctological surgery and allows for nontraumatic anal dilation to avoid damage to the specimen. Pathology analysis revealed a complete R0 resection of adenoma with high grade dysplasia.

**Video 1** Exposure of a large stalk vessel during dissection of a giant pedunculated colorectal polyp.


**Fig. 1:**
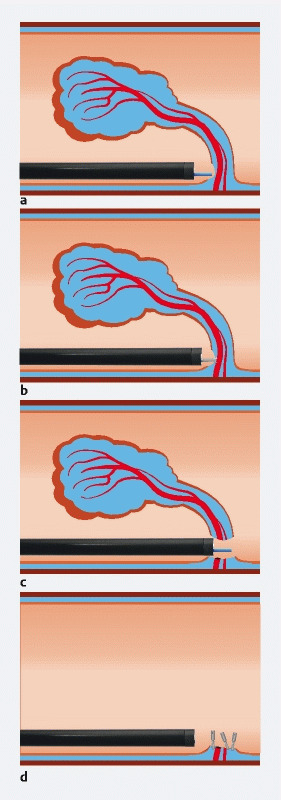
Schematic representation of the procedure. **a** Incision of the stalk mucosa. **b** Isolated vessels are coagulated using hemostatic forceps. **c** After cutting the vessels, the dissection is completed. **d** The defect is then closed with hemostatic clips.

In the context of global expansion of ESD, this technique, when mastered, could be used for the resection of giant pedunculated colonic polyps, as it allows targeted preventive hemostasis of the vessels and overcomes the problem of positioning a detachable snare around a head larger than 25 mm.

Endoscopy_UCTN_Code_TTT_1AQ_2AD

## Notes

Endoscopy E-Videos

https://eref.thieme.de/e-videos
*E-Videos* is an open access online section of the journal *Endoscopy* , reporting on interesting cases and new techniques in gastroenterological endoscopy. All papers include a high-quality video and are published with a Creative Commons CC-BY license. Endoscopy E-Videos qualify for HINARI discounts and waivers and eligibility is automatically checked during the submission process. We grant 100% waivers to articles whose corresponding authors are based in Group A countries and 50% waivers to those who are based in Group B countries as classified by Research4Life (see: https://www.research4life.org/access/eligibility/ ). This section has its own submission website at https://mc.manuscriptcentral.com/e-videos
